# MXene boosted MOF-derived cobalt sulfide/carbon nanocomposites as efficient bifunctional electrocatalysts for OER and HER†

**DOI:** 10.1039/d4na00290c

**Published:** 2024-04-26

**Authors:** Komal Farooq, Maida Murtaza, Zhuxian Yang, Amir Waseem, Yanqiu Zhu, Yongde Xia

**Affiliations:** a Department of Chemistry, Quaid-i-Azam University Islamabad 45320 Pakistan; b Department of Engineering, Faculty of Environment, Science and Economy, University of Exeter Exeter EX4 4QF UK y.xia@exeter.ac.uk

## Abstract

The development of effective bifunctional electrocatalysts that can realize water splitting to produce oxygen and hydrogen through oxygen evolution reaction (OER) and hydrogen evolution reaction (HER) is still a great challenge to be addressed. Herein, we report a simple and versatile approach to fabricate bifunctional OER and HER electrocatalysts derived from ZIF67/MXene hybrids *via* sulfurization of the precursors in hydrogen sulfide gas atmosphere at high temperatures. The as-prepared CoS@C/MXene nanocomposites were characterized using a series of technologies including X-ray diffraction, gas sorption, scanning electronic microscopy, transmission electronic microscopy, energy dispersive spectroscopy, and X-ray photoelectron spectroscopy. The synthesized CoS@C/MXene composites are electrocatalytically active in both HER and OER, and the CSMX-800 composite displayed the highest electrocatalytic performance towards OER and HER among all the produced samples. CSMX-800 exhibited overpotentials of 257 mV at 10 mA cm^−2^ for OER and 270 mV at 10 mA cm^−2^ for HER. Moreover, it also possesses small Tafel slope values of 93 mV dec^−1^ and 103 mV dec^−1^ for OER and HER, respectively. The enhanced electrocatalytic performance of the MXene-containing composites is due to their high surface area, enhanced conductivity, and faster charge transfer. This work demonstrated that CoS@C/MXene based electrocatalyst has great potential in electrochemical water splitting for hydrogen production, thus reducing carbon emissions and protecting the environment.

## Abstract 2

MXene-containing cobalt sulfide/carbon nanocomposites derived from MXene/ZIF-67 precursors exhibited highly efficient enhanced bifunctional electrocatalytic performances both in HER and OER.

## Graphical abstract

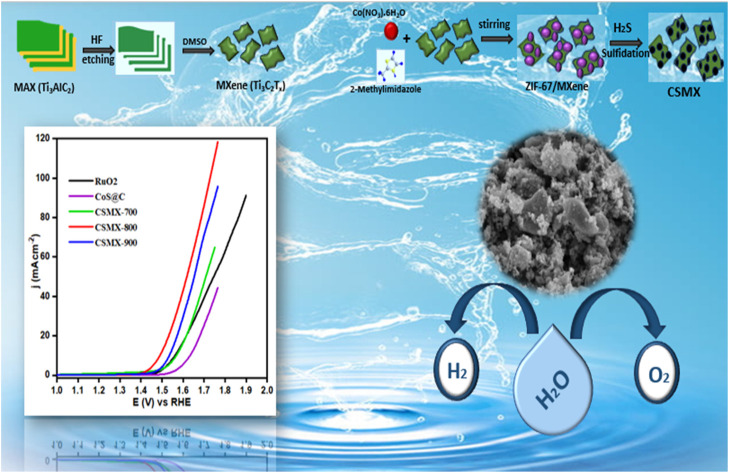

## 1. Introduction

The rapid expansion of global energy consumption demands novel and renewable energy sources. Fossil fuels are currently the predominant source of energy; however, continued reliance on fossil fuels raises severe environmental concerns, including greenhouse effect, global warming, and climate change,^1,2^ which have led to growing urgency to switch from hydrocarbon-based fossil fuels to cleaner and more sustainable sources of energy. Electrochemical water splitting provides a potential pathway to produce carbon-free and renewable hydrogen fuel, which can not only lower our reliance on fossil fuels but also offers a promising approach to achieve the transition to renewable energy sources in the future.^3,4^

Hydrogen evolution reaction (HER) at the cathode and oxygen evolution reaction (OER) at the anode are the two half processes in electrochemical water splitting. However, the efficiency of water electrolyzer is inevitably decreased by the slow kinetics on both electrodes. The potential for water splitting occurring in actual practice is much higher than the theoretical potential value of 1.23 V due to the need of overpotential to overcome the sluggish kinetics of the chemical reactions.^5^ Using appropriate electrocatalysts can speed up the reaction kinetics on both the electrodes and reduce their overpotentials to minimum values. The benchmark electrocatalysts are Pt-based materials for HER and IrO_2_ or RuO_2_ for OER; however, their high cost and the scarcity of these noble metal resources on earth restrict their widespread application.^6^ Therefore, it is crucial to explore earth-abundant, low-cost, stable and highly efficient functional electrocatalysts for water splitting.

Metal–organic frameworks (MOFs) have emerged as promising materials in electrochemical applications owing to their well-defined structures, tunable properties, high surface areas, and rich porosities.^7–9^ Zeolitic imidazole frameworks (ZIFs) are a sub-class of MOFs and have attracted significant interest in recent years owing to their potential applications in various fields including adsorption, separation, and catalysis. These materials consist of metal ions or metal clusters coordinated with imidazolate ligands to form porous, crystalline structures with tunable properties.^10,11^ Despite the high concentration level of metal ions in MOFs, the organic ligands, which serve as the coordination layer, totally block the connection between the metal ions, lowering the electrical conductivity and also the stability of MOFs, which inevitably affects the utilization of MOFs as electrocatalysts in harsh acidic or alkaline environments.^8,12^

MXene, discovered in 2011 by Gogotsi and co-workers, has attracted increasing attention due to its outstanding electrical conductivity (10^3^ to 10^4^ S cm^−1^),^13^ good hydrophilicity,^14^ high mechanical stability,^15^ low cost and environmental friendliness. MXene is a two-dimensional transition-metal carbide or nitride which can be represented as M_*n*+1_X_*n*_T_*x*_, where M is a transition metal such as Ti, Cr, Mn, Zr, *etc.*, X may be either nitrogen or carbon or a combination, *n* varies between 1 and 3, and T_*x*_ denotes hydrophilic surface functional groups such as =O, –OH, and –F.^16,17^ MXene has the valuable merits of high surface area, good electrical conductivity, accelerated charge transport kinetics and robust interfacial interaction.^18^ As a result, MXene is potentially an ideal material to be coupled with a MOF to produce efficient electrocatalysts for water splitting.^19^

Recent studies indicated that sulfurization of ZIF-67 derived cobalt-based materials demonstrated effectively enhanced electrocatalytic performance for water splitting. A straightforward metal–organic framework-engaged technique was described by Guo *et al.* to produce hollow Co_3_S_4_@MoS_2_ heterostructures that were utilized as efficient bifunctional catalysts for the generation of both H_2_ and O_2_*via* water splitting.^20^ Chen *et al.* presented a facile method for the synthesis of Ni–Co based sulfide/carbon nanocomposites by a one-step simultaneous sulfurization and carbonization of Ni-substituted ZIF-67.^21^ The Ni–Co based sulfide/carbon composites exhibited superior OER activity, more favourable kinetics, and longer durability as well as enhanced HER activities due to the Ni substitution, the high porosity, the homogeneous dispersion of active components and the effect of N,S-co-doping in carbons. Moreover, Huang *et al.* successfully developed a new approach to produce defect-rich and ultra-fine bimetallic Co–Mo sulfide/carbon composites from polyoxometalates@ZIF-67@polydopamine nanocubes *via* carbonization/sulfurization which are highly active for both HER and OER.^22^ Zhao *et al.* synthesized Mo–CoS_2_ nanoparticles generated from ZIF-67 and applied them in a hierarchically porous carbon hollow sphere for effective overall water splitting.^23^ WS_2_/Co_1−*x*_S@ porous carbon composites were also generated utilizing *in situ* synthesized phosphotungstic acid@ZIF-67 as precursors. The produced bimetallic tungsten-cobalt sulfide-based heteroatom doped porous carbon nanocomposites not only exhibited prominent improvement in electrocatalytic activities towards both HER and OER, but also showed increased stability in electrocatalytic performance due to the effective prevention of the agglomeration of metal sulfide particles by the *in situ* formed porous carbon matrix.^24^ Similarly, Deng *et al.* generated graphene-reinforced cobalt sulfide/carbon nanocomposites from *in situ* synthesized graphene/metal–organic framework composites as an effective multifunctional electrocatalyst.^25^ The produced CoS@C/10Graphene composite not only showed excellent electrocatalytic activity toward ORR with superior durability, but also exhibited good performance for OER and HER.

Built on the aforementioned analysis, in this work we report a versatile approach to produce MXene (Ti_3_C_2_) decorated CoS@C nanocomposites derived from the sulfurization of ZIF-67/MXene composite precursors in hydrogen sulfide (H_2_S) gas atmosphere at high temperatures. The obtained CoS@C/MXene composites are efficient and stable bifunctional electrocatalysts with enhanced electrochemical performance towards both OER and HER. The synergistic effect between the CoS@C and MXene in the composites not only endows good electrical conductivity and high surface area, but also provides appropriate active sites and superior catalytic performance towards water splitting.

## 2. Experimental

### 2.1 Materials

99% MAX phase (Ti_3_AlC_2_) was purchased from Nanoshel, UK. 48% hydrofluoric acid (HF), dimethyl sulfoxide (DMSO), 99% cobalt(ii) nitrate hexahydrate (Co(NO_3_)_2_·6H_2_O), and 99% 2-methylimidazole (C_4_H_6_N_2_, abbreviated as MeIM) were bought from Maklin Chemicals. 5 wt% Nafion solution and 20% Pt/C and 99.9% RuO_2_ catalysts were purchased from Sigma-Aldrich.

### 2.2 Synthesis of MXene

2 g MAX (Ti_3_AlC_2_) powder was mixed with 20 mL 48% HF and magnetically stirred at room temperature for 24 hours to selectively etch the Al layers in the MAX. The resulting suspension was then centrifuged and washed with distilled water several times until the pH was adjusted to neutral. The collected precipitate was freeze dried for 24 hours to get MXene Ti_3_C_2_T_*x*_.^26^

1 g as-prepared MXene powder was dispersed in 30 mL DMSO and magnetically stirred for 20 hours at room temperature in nitrogen atmosphere. The resulting suspension was centrifuged and washed several times with distilled water to get the intercalated Ti_3_C_2_T_*x*_. The residue was then dispersed in distilled water and ultrasonicated for 30 minutes at room temperature to exfoliate it into few-layer sheets, which were subsequently centrifuged and freeze dried to obtain few-layer Ti_3_C_2_T_*x*_ nanosheets.

### 2.3 Synthesis of ZIF-67 and ZIF67/MXene composites

75 mL of 0.1 M cobalt nitrate hexahydrate (Co(NO_3_)·6H_2_O) methanol solution and 75 mL of 0.8 M 2-methylimidazole methanol solution were combined and magnetically stirred for 1 hour at room temperature.^27^ The resultant mixture was then centrifuged, washed three times with ethanol and kept in the oven for drying at 80 °C overnight.

Three different ZIF67/MXene composites were prepared by altering the amounts of MXene (0.05, 0.1 and 0.2 g). The desired amount of MXene powder was dispersed in 20 mL of methanol using an ultrasonicator. 0.1 M cobalt nitrate (75 mL) methanol solution was added into the MXene suspension and stirred for 15 minutes. Then, 0.8 M 2-methylimidazole (75 mL) methanol solution was added dropwise into the MXene-containing solution, and the mixture solution was kept on magnetic stirring for about 2 hours at room temperature. The resulting mixture was then centrifuged, washed with ethanol several times and dried at 80 °C overnight in an oven.

### 2.4 Sulfurization of ZIF67/MXene composites

The as-synthesized ZIF67/MXene precursor sample was transferred to a ceramic boat and placed inside a tube furnace. The tube furnace was first flushed with argon gas for 1 hour and then heated to various desired temperatures (usually 700, 800 or 900 °C) under the continuous flow of pure argon gas with a heating ramp rate of 5 °C min^−1^. Once the desired temperature was reached, hydrogen sulfide (H_2_S) gas was introduced into the tube at a flow rate of 50 mL min^−1^, and the system was maintained at the target temperature for 1 hour. After completion, the H_2_S gas valve was closed, and the furnace was cooled to room temperature in an argon atmosphere. The products obtained from ZIF67/MXene-0.1 precursor at sulfurization temperatures of 700, 800 and 900 °C were named CoS@C/MXene-700 (CSMX-700), CoS@C/MXene-800 (CSMX-800) and CoS@C/MXene-900 (CSMX-900), respectively. The composites from the sulfurization of the ZIF67/MXene precursors with variable amounts of MXene (0.05 g, 0.1 g and 0.2 g) at 800 °C for 1 hour in H_2_S atmosphere were labelled as CSMX800-0.05, CSMX800-0.1 and CSMX800-0.2, respectively. For comparison, pure ZIF-67 and MXene samples were also sulfurized in H_2_S gas atmosphere at 800 °C for 1 hour and the obtained products were termed CoS@C and MXS, respectively.

### 2.5 Materials characterization

Powder X-ray diffraction (XRD) measurements were conducted on a Bruker D8 Advance X-ray diffractometer using Cu-Kα radiation with a scan rate of 5° min^−1^ and a step size of 0.02°. A Quantachrome Autosorb-iQ gas sorptometer was used to perform N_2_ gas sorption analysis. The surface areas of the samples were determined based on Brunauer–Emmett–Teller (BET) method using the adsorption data in the partial pressure (*P*/*P*_0_) range of 0.05–0.2 and the total pore volumes were calculated from the amount of adsorbed N_2_ at *P*/*P*_0_*ca.* 0.99. SEM images of the samples were taken using a Philips XL-30 in high vacuum mode with an acceleration voltage of 20 kV. Samples were sputtered with gold to reduce the effect of charging. The TEM images were captured using a JOEL 2100 at an acceleration voltage of 100 kV. Samples were first dispersed by sonication in absolute ethanol for 10 min and then deposited on a holey carbon copper grid. X-ray photoelectron spectroscopy (XPS) was carried out on a Kratos Axis Ultra system with a monochromated Al Kr X-ray source operated at 10 mA emission current and 15 kV anode potential.

### 2.6 Electrochemical measurements

Electrochemical measurements were carried out on a CHI660E workstation paired with a rotating disk electrode (RDE) system. The computer controlled potentiostat contained a three-electrode setup with Pt wire as the counter electrode, Hg/HgO as the reference electrode and a glassy carbon electrode (3 mm) coated with the testing material as the working electrode. 1.0 mg of synthesized sample was dispersed in a mixed solution containing 10 μL of water/ethanol solution, 80 μL of isopropanol and 10 μL of 5 wt% Nafion solution which was sonicated for about 7 hours to get a homogeneous slurry. This prepared catalyst ink was drop-cast on freshly polished glassy carbon and allowed to dry in air at room temperature. For OER measurements, 1 M KOH solution, purged with oxygen gas for half an hour before running the measurement, was used. For HER measurements, non-purged 0.5 M H_2_SO_4_ solution was utilized at room temperature. Using the Nernst equation, all the measured potentials were transformed to the reversible hydrogen electrode (RHE) scale: *E*_(RHE)_ = *E*^0^ + 0.098 + 0.0592 × pH. All the linear sweep voltammetry (LSV) curves were obtained at a scan rate of 5 mV s^−1^ and calibrated with 90% *iR* compensation.

## 3. Results and discussion

### 3.1 Characterization of synthesized materials

**Fig. 1:**
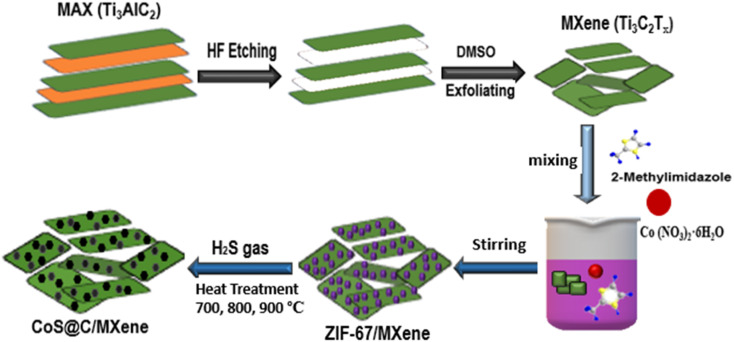
Schematic illustration of the synthesis of CoS@C/MXene nanocomposites at different temperatures.

A schematic diagram of the material synthesis process is presented in Fig. 1. MXene (Ti_3_C_2_T_*x*_) was first produced from MAX (Ti_3_AlC_2_) *via* etching in HF solution and exfoliation in DMSO solvent. Then, the parental ZIF67/MXene precursor was synthesized *via* a simple one-pot hydrothermal method by stirring a mixture solution of 2-methylimidazole, Co(NO_3_)_2_ and the desired amount of MXene in methanol at room temperature for 2 hours. The as-synthesized ZIF67/MXene was then subjected to high-temperature pyrolysis in a H_2_S/argon atmosphere to generate the target cobalt sulfide@carbon/MXene nanocomposites.

The detailed structures of the prepared precursor samples were acquired by powder X-ray diffraction (XRD) analysis. As shown in Fig. S1,† the XRD pattern of the MXene (Ti_3_C_2_T_*x*_) sheets lacks the diffraction peak for the (104) plane of the MAX phase at 2*θ* of 38.8°, which indicates that the Al layers in MAX were successfully removed. The successful synthesis of ZIF-67 was confirmed by the matching of its XRD patterns with those in the literature,^28^ which suggests the excellent purity and crystallinity of the as-synthesized ZIF-67. The XRD patterns of the ZIF67/MXene precursor represent an overlapping of the ZIF-67 and MXene phases, which implies that the crystallinity of ZIF-67 was not affected by the addition of MXene in the composites, as the 2*θ* values and intensities of the main diffraction peaks for both ZIF-67 and ZIF67/MXene are almost identical (Fig. S1†).

**Fig. 2:**
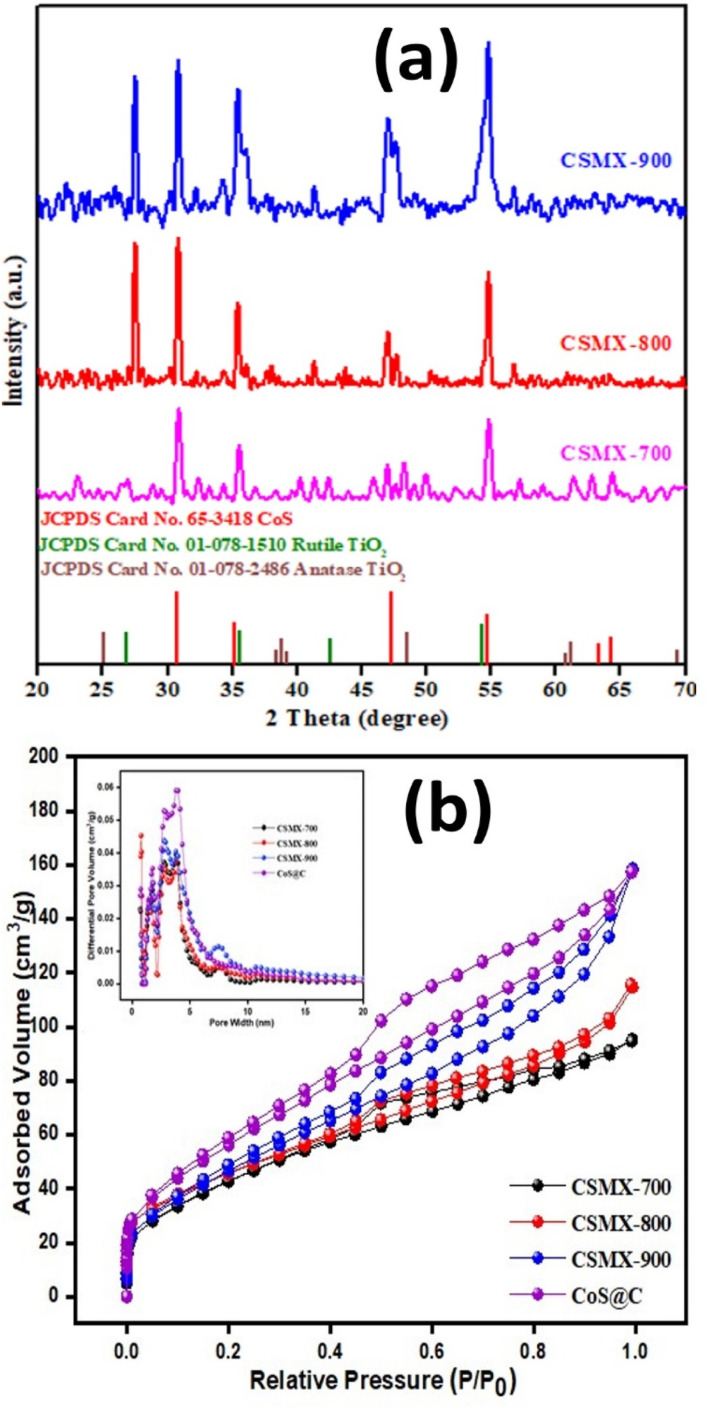
(a) Powder XRD patterns and (b) N_2_ sorption isotherms of CSMX-700, CSMX-800 and CSMX-900 composites. The inset in (b) is the corresponding pore size distribution curves of CSMX-700, CSMX-800, CSMX-900 and CoS@C.

Fig. 2a presents the XRD patterns of the samples prepared under different sulfurization temperatures. All the samples showed several diffraction peaks at 2*θ* of 30.6°, 35.3°, 46.9° and 54.4°, belonging to the (100), (101), (102) and (110) planes of the CoS phase according to JCPDS reference card number 65-3418.^29^ The peak broadening was probably due to the small particle sizes and poor degree of crystallization of CoS. The intensities of the main diffraction peaks increase with sulfurization temperature, suggesting that higher sulfurization temperatures led to a higher degree of crystallinity in the produced CSMX samples. No XRD peaks from anatase or rutile phases were found in CSMX-700, maybe due to the low content of MXene in the sample. However, in the CSMX-800 and CSMX-900 composites obtained at higher sulfurization temperatures of 800 and 900 °C, the presence of the rutile phase was clearly observed at 2*θ* values of 27.4, 36.1 and 54.4°, corresponding to the (110), (101) and (211) planes of rutile (JCPDS reference 01-078-1510).^30^ These observations are in accordance with the elemental analysis by EDX, as shown in SEM analysis below.

The textural properties, including specific surface area, pore volume and pore size distribution, of the produced samples were evaluated by N_2_ gas sorption at liquid nitrogen temperature. The N_2_ sorption isotherms and pore size distribution curves of the samples CSMX-700, CSMX-800, CSMX-900 and CoS@C are displayed in Fig. 2b. The calculated Brunauer–Emmett–Teller (BET) surface areas of CoS@C, CSMX-700, CSMX-800 and CSMX-900 are 211, 160, 166, and 172 m^2^ g^−1^, respectively, and the pore volumes are in the range of 0.13–0.22 cm^3^ g^−1^ (Table S1†). It seems that high sulfurization/carbonization temperatures led to higher specific surface areas and pore volumes, possibly due to the full carbonization of the precursor ZIF67/MXene at higher temperatures. Obviously, the *in situ* development of porous carbon during the sulfurization/carbonization process is the one of the reasons for the observed increased textural properties of the composites. As shown in the inset of Fig. 2b on the pore size distribution curves derived from the adsorption branch of the N_2_ isotherms, all the CSMX and CoS@C composites exclusively exhibited both micropores centered at 1.6 nm and mesopores in the range of 2.2–4.3 nm, suggesting similar porosities in these composites.

**Fig. 3:**
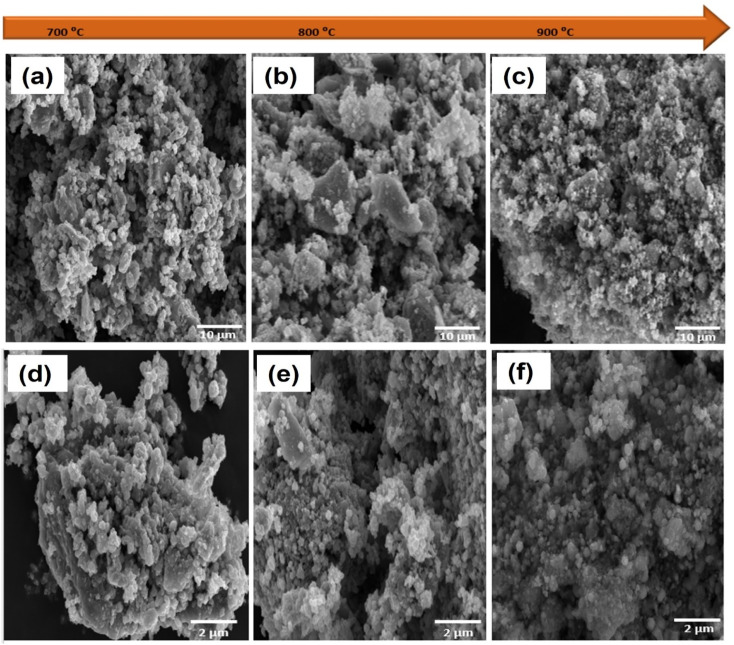
SEM images of (a) CSMX-700, (b) CSMX-800 and (c) CSMX-900 at 10 μm and (d) CSMX-700, (e) CSMX-800 and (f) CSMX-900 at 2 μm.

To investigate the surface topography of the prepared samples, scanning electron microscopy (SEM) was carried out. As shown in Fig. S2,† MXene sheets were successfully synthesized by the etching and exfoliation processes.^31^ ZIF-67 exhibits cubic and tetragonal shapes, which agrees well with the previous report in literature (Fig. S2c†).^27^ The SEM image of the ZIF-67/MXene composite shows that the MXene sheets were covered and overlaid with ZIF-67 (Fig S2d†). The negative charges in Ti_3_C_2_T_*x*_ sheets may attract the Co^2+^ ions in ZIF-67 to their surface *via* electrostatic interaction, and the exposed N atoms on the surface of ZIF-67 may readily form hydrogen bonds with the functional groups –F and –OH on the surface of Ti_3_C_2_T_*x*_.^19^ As illustrated in Fig. 3, the surface morphologies of the CSMX-700, CSMX-800 and CSMX-900 composites derived from the parental ZIF-67/MXene-0.1 composite were investigated by SEM. After sulfurization treatment in H_2_S atmosphere at high temperatures, many irregular particles were observed in the obtained CoS@C/MXene composites, which may be due to the fact that ZIF-67 on Ti_3_C_2_T_*x*_ reacted with hydrogen sulfide at high temperature and these irregular particles then agglomerated to form dense clumps with rough surfaces. As the sulfurization temperature increases from 700 °C to 900 °C, multiple small, spherical particles can be observed in the CSMX composites. EDX elemental analysis showed that Co, C, S, Ti and O elements (Fig. S3†) exist in all the CSMX composites with slightly different elemental contents depending on their heat treatment temperatures.

**Fig. 4:**
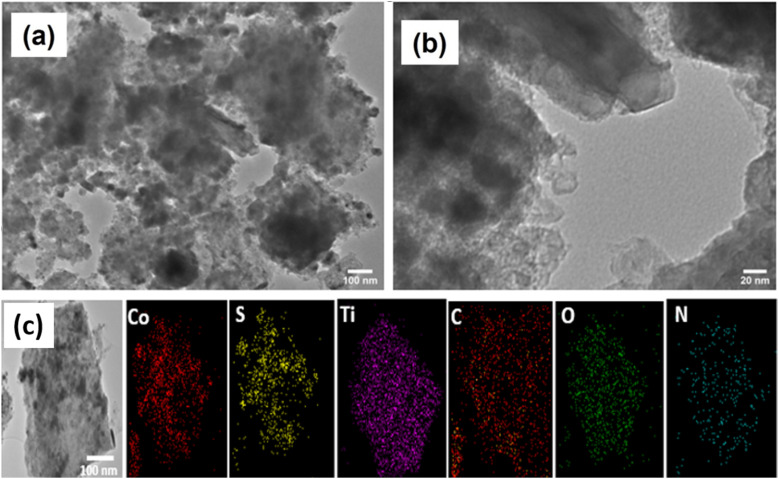
Representative TEM images of CSMX-800 at (a) low magnification of 100 nm and (b) high magnification of 20 nm. (c) Elemental mapping of sample CSMX-800.

High resolution transmission electron microscopy (TEM) images of the representative CSMX-800 nanocomposite are presented in Fig. 4. The TEM images revealed that CoS particles are wrapped around, adhered to and even inside the Ti_3_C_2_T_*x*_ sheets. As a result, the crystallinity of CoS decreases and the overall morphologies of the composite particles become asymmetrical. Moreover, a few individual Ti_3_C_2_T_*x*_ clusters can be clearly observed. These clusters may eventually form a composite material with CoS particles through hydrogen bonding and/or other interactions. From the selected area of TEM image of CSMX-800, the elemental mapping images of different elements were acquired and are shown in Fig. 4c. Elements including Co, C, S, N, Ti, and O are presented in the elemental mappings and all elements show images similar to the selected TEM image, indicating not only the homogeneous distribution of these elements in the composites, but also the evenly distributed CoS nanoparticles on MXene sheets in the composite. The presence of O in sample CSMX-800 is consistent with the SEM-EDX and XRD results and confirms the formation of TiO_2_ in the composite.

X-ray photoelectron spectroscopy (XPS), a powerful surface technique that can identify elements and their states in samples, was performed. The wide-range XPS elemental survey of representative samples CSMX-800 and CoS@C, shown in Fig. S4,† exhibits the presence of Co, C, N, O and S in both samples, while the presence of a Ti peak is observed in the CSMX-800 sample.

**Fig. 5:**
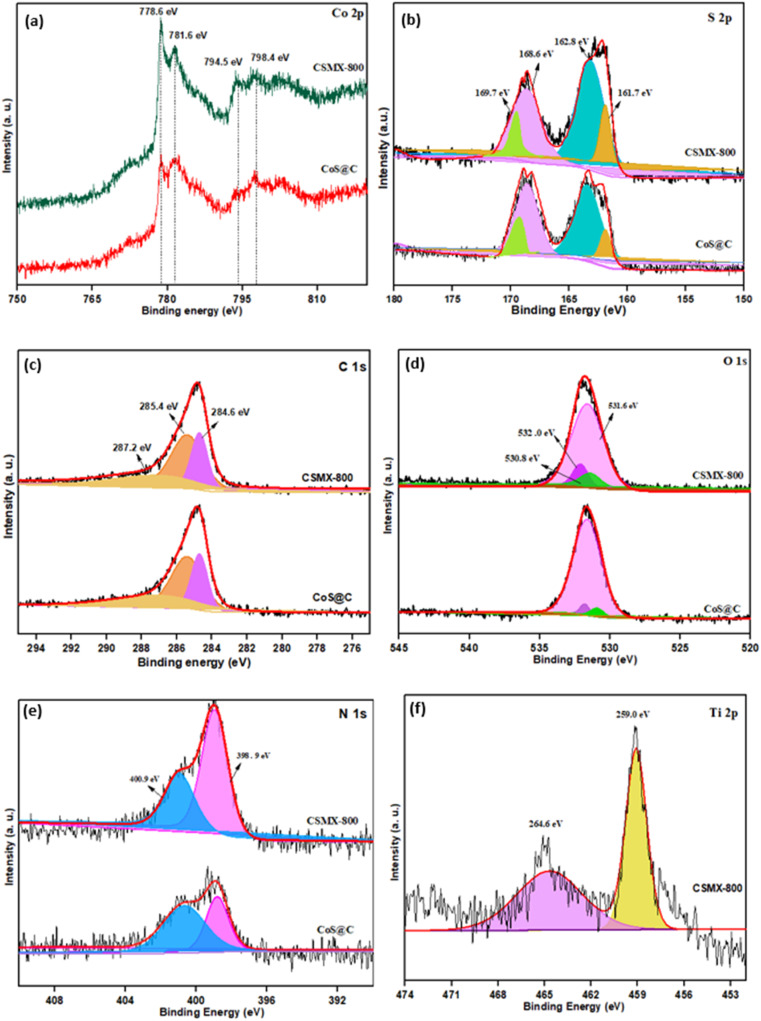
High-resolution XPS spectra of (a) Co 2p, (b) S 2p, (c) C 1s, (d) O 1s, (e) N 1s and (f) Ti 2p for CSMX-800 and CoS@C.

As shown in Fig. 5a, the XPS spectra of Co 2p in both samples CSMX-800 and CoS@C show two sets of spin–orbit doublet peaks. The peaks at approximately 781.6 and 798.4 eV are from 2p_3/2_ and 2p_1/2_ of Co 2p, respectively, which are characteristic of the Co^2+^ oxidation state in the surface species^32^ and belong to the Co–S bond due to the formation of cobalt sulfide.^33^ The peaks at around 778.6 and 794.5 eV can be ascribed to 2p_3/2_ and 2p_1/2_ of Co 2p with a higher oxidation state, suggesting that mixed oxidation states existed in both the CSMX-800 and CoS@C samples, which is consistent with a previous report.^34^ The deconvoluted S 2p spectra, as presented in Fig. 5b, reveal two peaks at binding energy values of 161.7 and 162.8 eV, which correspond to the S 2p_5/2_ and S 2p_3/2_ doublets with a binding energy separation of 1.1 eV, due to the S^2−^ species in the formed cobalt sulfide.^35^ Moreover, the doublet peaks at binding energies of 168.6 and 169.7 eV are assigned to the S 2p_5/2_ and S 2p_3/2_ of oxidized S species, such as sulfate groups, which could be due to the partial oxidation of sulfur in air or oxygen-containing species in MXene or intermediate products formed during the transformation of the ZIF-67/MXene precursor in the H_2_S atmosphere.^21,22^

The XPS spectra of C 1s can be deconvoluted into three individual peaks at 284.6, 285.4 and 287.2 eV, as shown in Fig. 5c. The deconvoluted peak at 284.6 eV is the contribution from C–C or C=C, while the peak at 285.4 may derive from the single and/or double bonded C–N species, and the deconvoluted peak at 287.2 eV is attributed to C=O or C–O species.^21^ The presence of N species in the produced nanocomposites is confirmed by both the XPS element survey and SEM-EDX. The N 1s spectra show two peaks at binding energy values of ∼398.9 and ∼400.9 eV (Fig. 5e) which can be attributed to pyridinic-N and pyrrolic-N, respectively.^36^ The deconvoluted O 1s with binding energy at 531.6 eV is allocated to O^2−^ in metal oxides such as TiO_2_ or Co_*x*_O_*y*_, the peak at a binding energy of 532.0 eV is for carbonyl group C=O, and the most intense peak at 530.8 eV is due to the metal-bound hydroxyl groups on the surface of the sample. In the XPS spectra of Ti (Fig. 5f), two peaks are clearly observed. The one at a binding energy of 459.0 eV is attributed to Ti 2p_3/2_, while the one at a binding energy of 464.6 eV corresponds to Ti 2p_1/2_.

### 3.2 Electrochemical performance of CoS@C/MXene composites

**Fig. 6:**
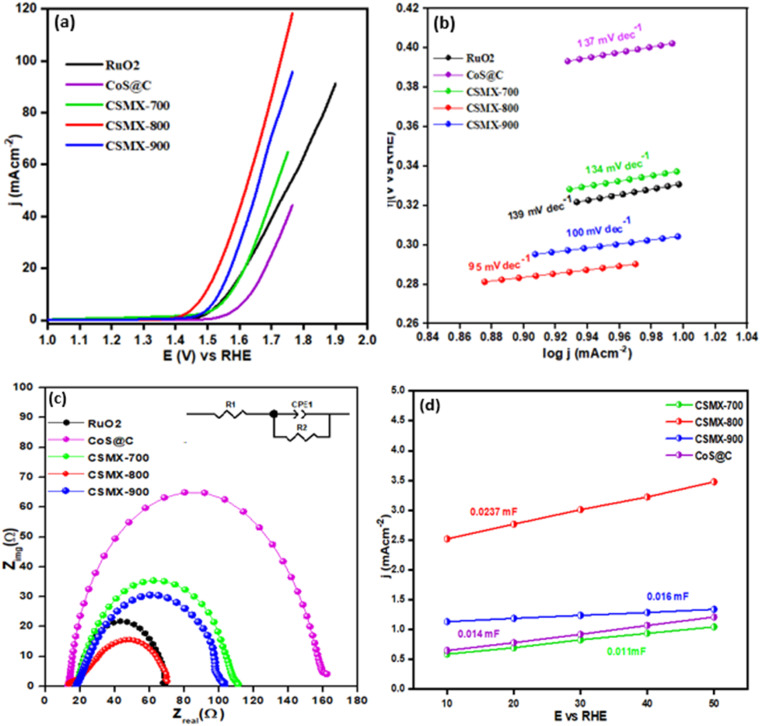
(a) OER linear sweep voltammograms (LSV), (b) Tafel slopes and (c) EIS spectra in the range of 10^2^ to 10^6^ Hz for samples RuO_2_, CSMX-700, CSMX-800, CSMX-900 and CoS@C. (d) Double layer capacitance of composites CSMX-700, CSMX-800, CSMX-900 and CoS@C.

**Fig. 7:**
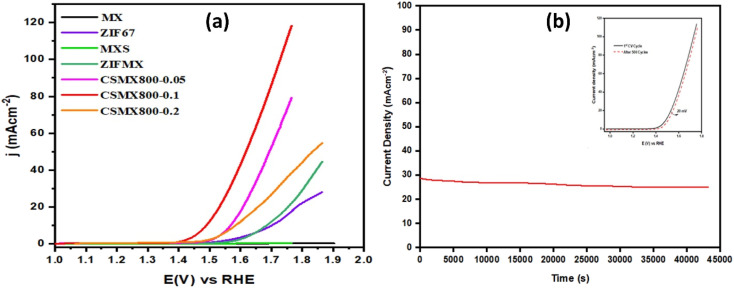
(a) OER LSV curves of CSMX with variable MXene content (samples CSMX800-0.05, CSMX800-0.1 and CSMX800-0.2) and relevant reference samples MXene (MX), ZIF67, MXS and ZIF67/MXene (ZIFMX). (b) Chronoamperometric response test (*i*–*t*) of CSMX-800 for 12 hours. The inset is the OER LSV curves of 1st CV and after 500 CV cycles.

The electrocatalytic activities of the produced composites towards oxygen evolution reaction (OER) were examined in an O_2_ saturated 1 M KOH electrolyte solution at 1600 rpm with a scanning rate of 5 mV s^−1^ and the results are presented in Fig. 6a. From the LSV polarization curves, it is clear that CSMX-800 shows the highest oxygen evolution catalytic activity, with an overpotential of 257 mV at a current density of 10 mA cm^−2^, which is superior to those of RuO_2_@GCE (331 mV), CoS@C (403 mV), ZIFMX (456 mV), CSMX-700 (339 mV) and CSMX-900 (305 mV) at the same current density, as shown in Table S2.†Fig. 6a also suggests that the sulfurization temperatures of the ZIF67/MX precursors play an important role in their OER performance, and sulfurization at 800 °C (sample CSMX-800) leads to the best performing CSMX composite in OER with remarkably decreased overpotential. The OER results in Fig. 6a and 7a also clearly indicate that the electrocatalytic activities of the composites CoS@C/MXene are much higher than those of the bulk ZIF-67 and MXene precursors as well as their sulfurized composites.

The Tafel slope (shown in Fig. 6b) of CSMX-800 is only 95 mV dec^−1^, which is much smaller than those of RuO_2_ (139 mV dec^−1^), CoS@C (137 mV dec^−1^), ZIFMX (183 mV dec^−1^), CSMX-700 (134 mV dec^−1^) and CSMX-900 (100 mV dec^−1^), suggesting much faster OER kinetics for CSMX-800 compared with other samples. This observation indicates that the introduction of MXene into ZIF-67 precursor followed by sulfurization dramatically improves the electrocatalytic activity of the composites.

Electrochemical impedance spectroscopy (EIS) measurements between 10^2^ and 10^6^ Hz (as shown in Fig. 6c) were carried out to compare the real and imaginary components of the Nyquist plots and analyze the kinetics of the various prepared composites. Among all the studied catalysts, sample CSMX-800 showed the smallest half-circle, indicating that this catalyst possesses the lowest resistance to charge transfer (*R*_ct_). As a result, sample CSMX-800 has an enhanced charge transfer capacity which accelerates the process of oxygen evolution. Cyclic voltammograms at different scanning rates in the non-faradaic region (shown in Fig. S5†) were obtained to determine the values of double layer capacitance (*C*_dl_). By plotting the anodic current against the scanning rates, the double layer capacitance *C*_dl_ is assessed from the determined slope of the linear regression (shown in Fig. 6d). CSMX-800 showed a *C*_dl_ value of 0.0237 mF, higher than those of CSMX-700, CSMX-900 and CoS@C. The higher value of *C*_dl_ suggests the presence of more catalytic active sites, higher surface area and, hence, better electrocatalytic activity, which is consistent with the OER results. The electrochemical surface area (ECSA) of a sample can be calculated using the following formula.ECSA = *C*_dl_/*C*_s_where *C*_s_ is the specific capacitance of the electrode and has a value of 0.02 mF cm^−2^ in 1 M KOH electrolyte. Obviously, the value of ECSA of an electrocatalyst has a direct relationship with the value of double layer capacitance *C*_dl_ of the electrode material: an electrocatalyst with a higher *C*_dl_ value also has higher ECSA and *vice versa*. The calculated value of ECSA for sample CSMX-800 is 1.185 cm^2^, which is higher than those of the CSMX-700, CSMX-900 and CoS@C samples. Another important parameter for the electrocatalytic activity of an electrocatalyst is the roughness factor (*R*_f_) of the electrode's surface, which can be computed using the ratio of ECSA and the geometric area of the electrode. As presented in Fig. 6d and Table S3,† the calculated value of the surface roughness factor for CSMX-800 is 16.92, which is also much higher than those of its counterparts CSMX-700, CSMX-900 and CoS@C.

In order to evaluate the effect of MXene content in the resulting CoS@C/MXene (CSMX) composites on their OER performance, ZIF67/MXene with different amounts of MXene contents were prepared and used as precursors to sulfurize/carbonize at 800 °C in H_2_S atmosphere. The OER performances of the obtained CoS@C/MXene composites with variable MXene contents and some relevant reference samples are presented in Fig. 7a. It is clear that the produced CoS@C/MXene composites with variable MXene contents exhibited much improved OER activities compared with those of pristine MXene or ZIF-67 and their sulfurized composites obtained at the same sulfurization/carbonization temperature of 800 °C in H_2_S atmosphere. Namely, MXene-containing CoS@C composites perform better in OER than the MXene-free CoS@C sample. Moreover, increasing the amount of MXene in the precursor ZIF67/MXene from 0.05 to 0.1 g increases the OER performance of the resulting CoS@C/MXene, maybe due to the increased electrical conductivity and good dispersion of active sites on the introduced 2D MXene sheets. However, on further increase of the MXene content in the precursor ZIF67/MXene to 0.2 g, the OER performance of the resulting CoS@C/MXene decreases, potentially due to the decrease in active sites as they become partially covered or blocked by the large introduced amount of MXene sheets. As a result, the CoS@C/MXene composite with an optimized amount of MXene (0.1 g) shows the best electrocatalytic performance towards OER among the fabricated composites under the same conditions.

The stability of an electrocatalyst is a crucial factor to determine the practical applications of the catalyst in the real world. The durability and stability of the CSMX-800 electrocatalyst in OER were evaluated by chronoamperometric response test (*i*–*t*) for a period of 12 hours at a voltage of 1.60 V (*vs.* RHE) in 1 M KOH aqueous solution (as shown in Fig. 7b). The catalyst CSMX-800 exhibits a current density of around 30 mA cm^−2^ and the current density was maintained without any observable loss during the whole measurement period of 12 hours. The catalyst upheld 93.3% of its initial current density after 12 hours of chronoamperometric measurements, implying great stability of the CSMX-800 electrocatalyst in OER. The stability of the catalyst was also evaluated by performing OER LSV scanning in the potential range of 1.2–1.8 V (*vs.* RHE) in 1 M KOH electrolyte solution. As evidenced by the inset in Fig. 7b, the OER LSV curve of the electrocatalyst CSMX-800 after 500 CV cycles is similar to its 1^st^ CV cycle, indicating that the electrocatalyst CSMX-800 exhibits excellent durability with a slight decline of potential of only 20 mV over 500 cycles. The increased electrocatalytic performance and the high stability of the CSMX-800 composite electrocatalyst is not only due to the exposure of more active sites by the 2D MXene material providing a conductive matrix for CoS, but also owing to the synergistic effect between CoS and MXene in the catalyst.

**Fig. 8:**
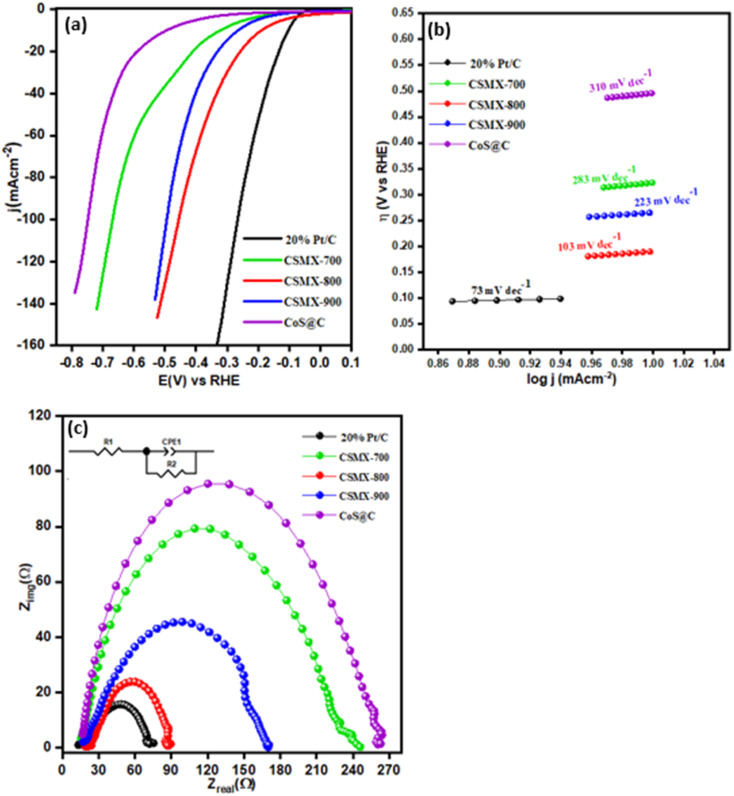
(a) HER linear sweep voltammograms (LSV), (b) Tafel slopes and (c) EIS spectra of 20% Pt/C, CSMX-700, CSMX-800, CSMX-900 and CoS@C.

The LSV curves of hydrogen evolution reaction (HER) for all samples, carried out in 0.5 M H_2_SO_4_ electrolyte solution on a three-electrode system, are presented in Fig. 8a. The samples 20% Pt/C and CoS@C were also included for comparison. The polarization curve of CSMX-800 shows an onset potential of −0.10 mV, which is lower than those of the other composites, including CSMX-900, CSMX-700 and CoS@C. To achieve the current density of 10 mA cm^−2^, an overpotential of 190 mV is required for CSMX-800, while the overpotential values are 265, 323 and 496 mA cm^−2^ for composites CSMX-900, CSMX-700, and CoS@C, respectively. It is evident that sample CSMX-800 exhibited the highest HER performance among all the prepared composites. However, the HER performances for all these composites are still lower than the benchmark 20% Pt/C sample. It is worth noting that all the CSMX composites exhibited much improved HER performance compared with that of sample CoS@C, and the enhanced HER performance of the CSMX composites is likely due to the high conductivity of the introduced MXene as well as the synergistic effect between MXene and the *in situ* formed cobalt sulfide/carbon.

The Tafel slopes of these samples are shown in Fig. 8b and were derived from their respective HER LSV curves. The CSMX-800 sample shows a Tafel slope of 103 mV dec^−1^, which is much lower than those of CoS@C, CSMX-900 and CSMX-700, but higher than that of the 20% Pt/C sample. These observations further reveal that CSMX-800 is a more effective electrocatalyst for HER than CoS@C, CSMX-900 and CSMX-700, but less effective than 20% Pt/C. In addition, EIS of the samples was also performed to further evaluate their catalytic ability, as shown in Fig. 8c. It is clear that sample CSMX-800 shows the least resistance to alternative current compared to the other synthesized samples; therefore, it exhibits an enhanced charge transfer capacity that can accelerate hydrogen evolution, which agrees well with the observed HER performance.

The HER and OER performances of the CSMX-800 catalyst were compared with those of certain sulfurized cobalt-based samples that were published in the literature. As presented in Table 1, the superior performance of the CSMX-800 catalyst over numerous other similar sulfurized cobalt-based catalysts is evident. The enhanced electrocatalytic activity of the CoS@C/MXene composite material, named CSMX composite, is due to the following reasons: (a) the presence of MXene and carbon components can significantly enhance electrical conductivity of the CoS@C/MXene composite, improving its electrocatalytic performance in both OER and HER. (b) The combination of cobalt sulfide, carbon, and MXene components can lead to synergistic effects, where each component contributes to the overall electrocatalytic activity and stability, thus resulting in the electrocatalytic performance of CoS@C/MXene composite being higher than those of each individual component. (c) Cobalt sulfide is an electrochemically active bifunctional component which provides catalytic active sites for both OER and HER. (d) The MXene component often possesses high surface area, which provides more exposed catalytic active sites for electrochemical reactions and thus enhances both the OER and HER performances of the composite electrocatalysts. Overall, CoS@C/MXene represents a promising electrocatalyst system with synergistic effects from its constituent materials, offering improved performance and good stability for both the oxygen and hydrogen evolution reactions.

**Table 1:** Comparison of OER and HER performances of different sulfurized cobalt-based electrocatalysts

Electrocatalyst	OER overpotential at 10 mA cm^−2^ (mV *vs.* RHE)	HER overpotential at 10 mA cm^−2^ (mV *vs.* RHE)	Reference
CSMX-800	257	190	This work
CoS@C/Graphene	450	280	25
CoMo-S/C	352	240	22
Co_3_S_4_@MoS_2_	330	210	20
Mo–CoS_2_/NC	296	158	23
CoS_2_(400)/N, S-GO	380	—	37
Co–CoO/Ti_3_C_2_-MXene	271	450	38
MXene@Ce-MOF	270	220	39
Co@N-CNTF	350	220	40

## 4. Conclusions

In summary, we present an effective and simple approach to produce cobalt sulfide embedded in S,N co-doped porous carbon and MXene nanocomposites. This involves heat treatment of ZIF-67/MXene precursor at different temperatures in H_2_S atmosphere. The produced CSMX-800 composite exhibits high catalytic activity and stability towards both OER and HER. The CSMX-800 electrocatalyst displays an overpotential of 257 mV at 10 mA cm^−2^ with a Tafel slope value of 95 mV dec^−1^ in OER and an overpotential of 190 mV at 10 mA cm^−2^ with a Tafel slope value of 103 mV dec^−1^ in HER, which are much lower than those of the sample without MXene (sample CoS@C). CSMX-800 also shows high stability and displays negligible current density loss during a 12 h running period. The robust connection between CoS particles and Ti_3_C_2_T_*x*_ resulting in enhanced electrical conductivity and effective electron transportation, the increased active sites provided by the MXene in the customized composite, and the synergistic effects between CoS and MXene are primarily responsible for the exceptional electrocatalytic performance of the CoS@C/MXene composites both in OER and HER. This work indicates that the introduction of MXene to ZIF-67 followed by sulfurization treatment at high temperature not only increases the electrical conductivity and electrochemical active surface area of the electrocatalysts, but also decreases the charge transfer resistance, benefiting the outstanding performance of the catalysts, which have great potential in electrochemical water splitting for green hydrogen production.

## Author contributions

Komal Farooq: investigation, methodology, data curation, and writing – original draft. Maida Murtaza: investigation, validation, and data curation. Zhuxian Yang: investigation, data curation, validation, and writing – review & editing. Amir Waseem: supervision, and writing – review & editing. Yanqiu Zhu: supervision, and writing – review & editing. Yongde Xia: supervision, conceptualization, methodology, validation, and writing – review & editing.

## Conflicts of interest

The authors declare no conflict of interest.

## Supplementary Material

NA-006-D4NA00290C-s001
