# Supplementary material for: MXene boosted MOF-derived cobalt sulfide/carbon nanocomposites as efficient bifunctional electrocatalysts for OER and HER
Source: Nanoscale Adv. 2024 Apr 26;6(12):3169–80. doi: 10.1039/d4na00290c (PMC11166099; doi:10.1039/d4na00290c)
Supplement: NA-006-D4NA00290C-s001 [file NA-006-D4NA00290C-s001.pdf]

Electronic Supplementary Material (ESI) for Nanoscale Advances.
This journal is © The Royal Society of Chemistry 2024

Supplementary materials

MXene boosted MOF-derived cobalt sulfide/carbon nanocomposites as efficient bifunctional electrocatalysts for OER and HER

Komal Farooq,[1,2] Maida Murtaza,[1] Zhuxian Yang,[2] Amir Waseem,[1] Yanqiu Zhu[2] and Yongde Xia[*2]

[1]*Department of Chemistry, Quaid-i-Azam University, Islamabad-45320, Pakistan*

[2]*Department of Engineering, Faculty of Environment, Science and Economy, University of Exeter, Exeter EX4 4QF, United Kingdom*. E-mail: y.xia@exeter.ac.uk

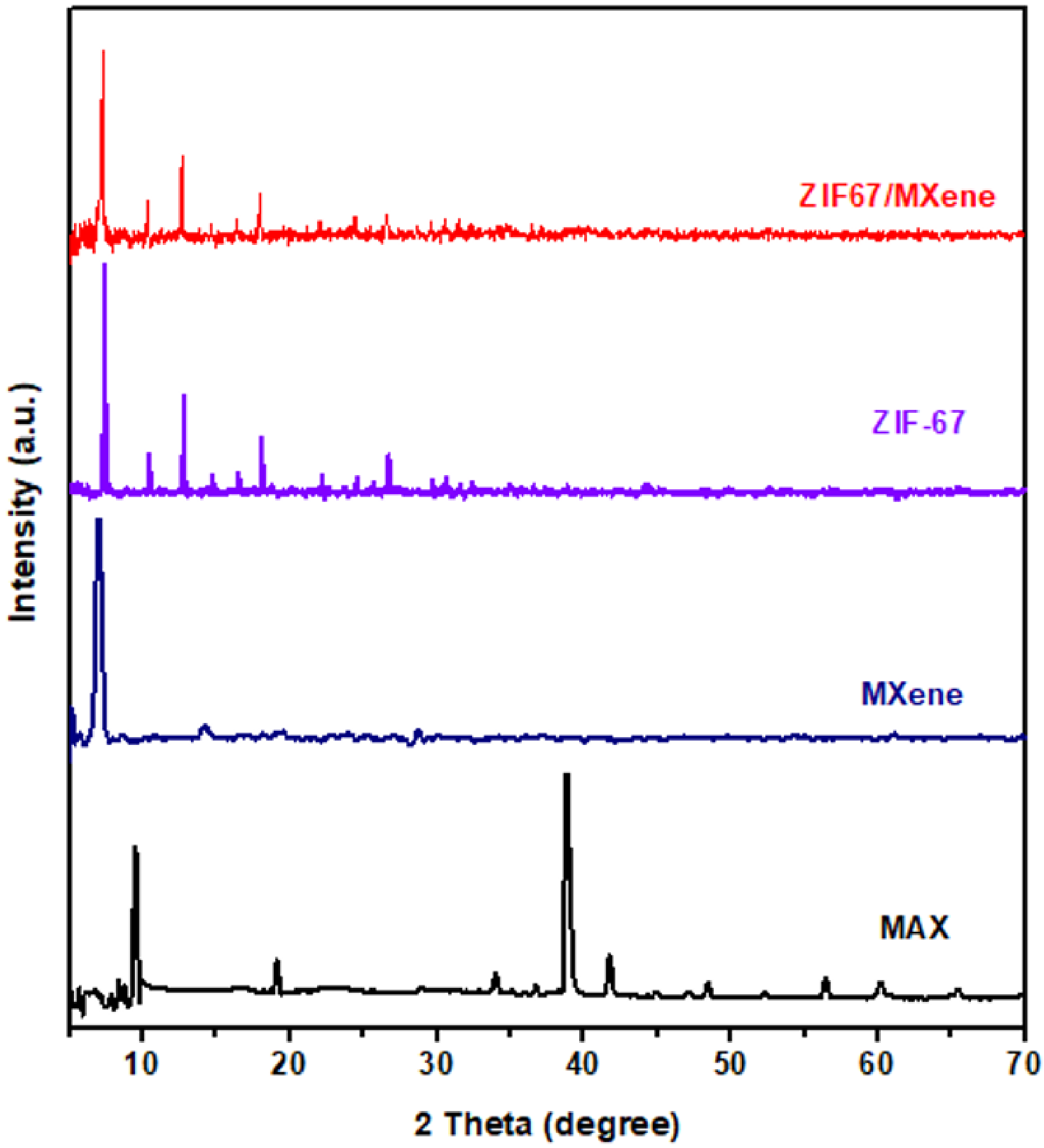

Fig. S1 Powder XRD patterns of MAX, MXene, ZIF-67 and ZIF67/MXene samples.

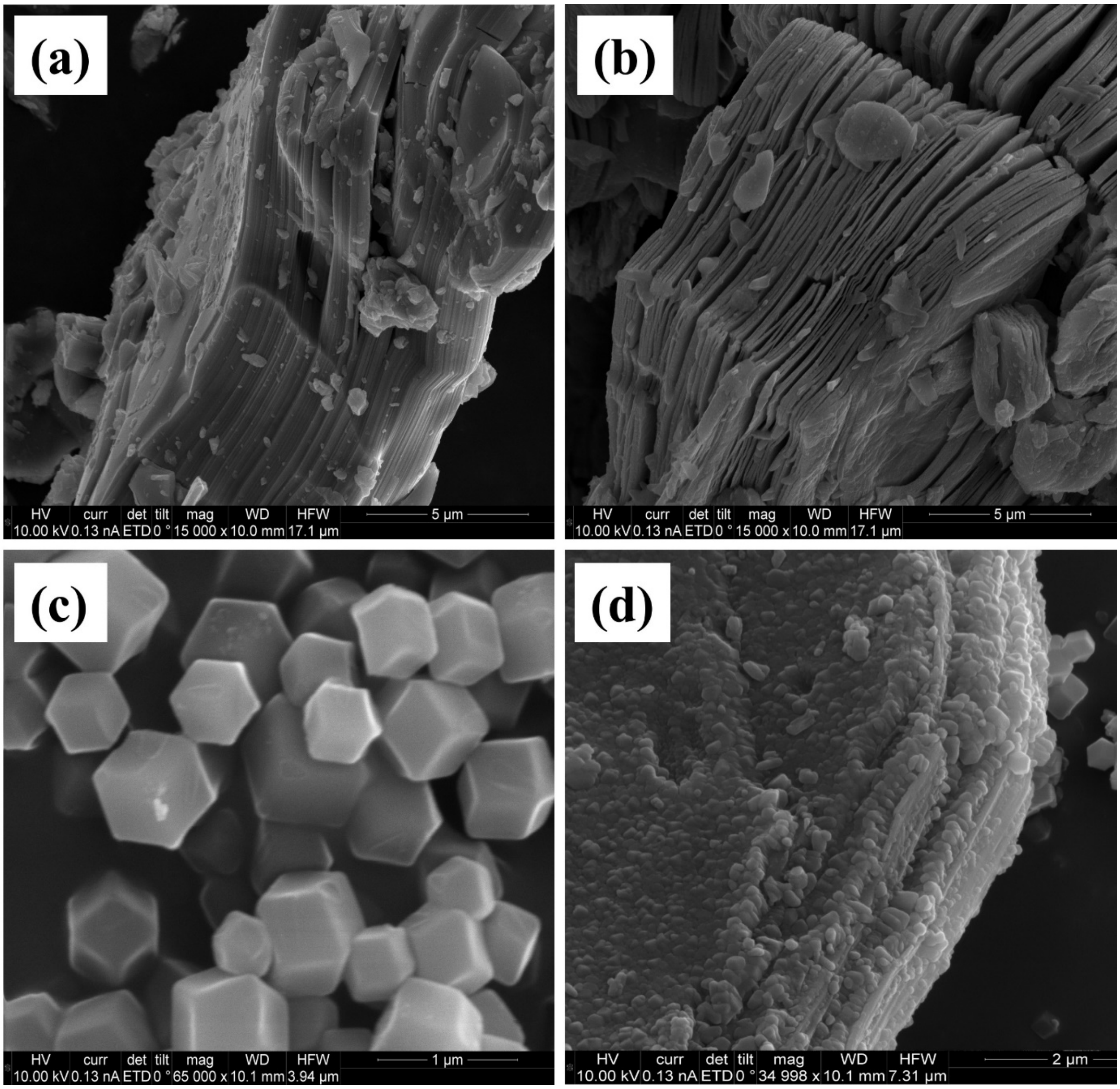

Fig. S2 FESEM images of (a) MAX; (b) MXene; (c) ZIF-67 and (d) ZIF67/MXene.

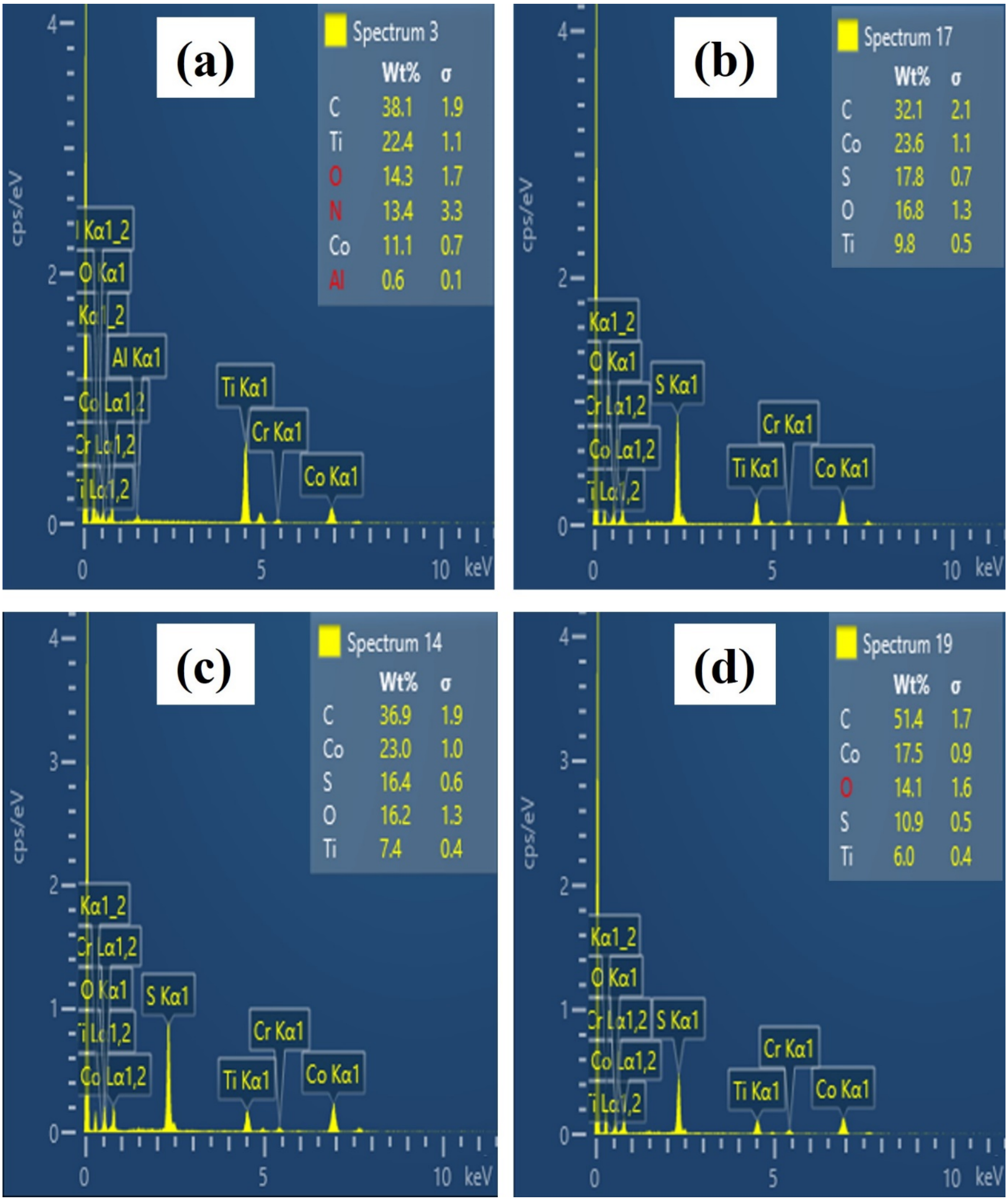

Fig. S3 EDX element analysis of (a) ZIF67/MXene; (b) CSMX-700; (c) CSMX-800 and (d) CSMX-900.

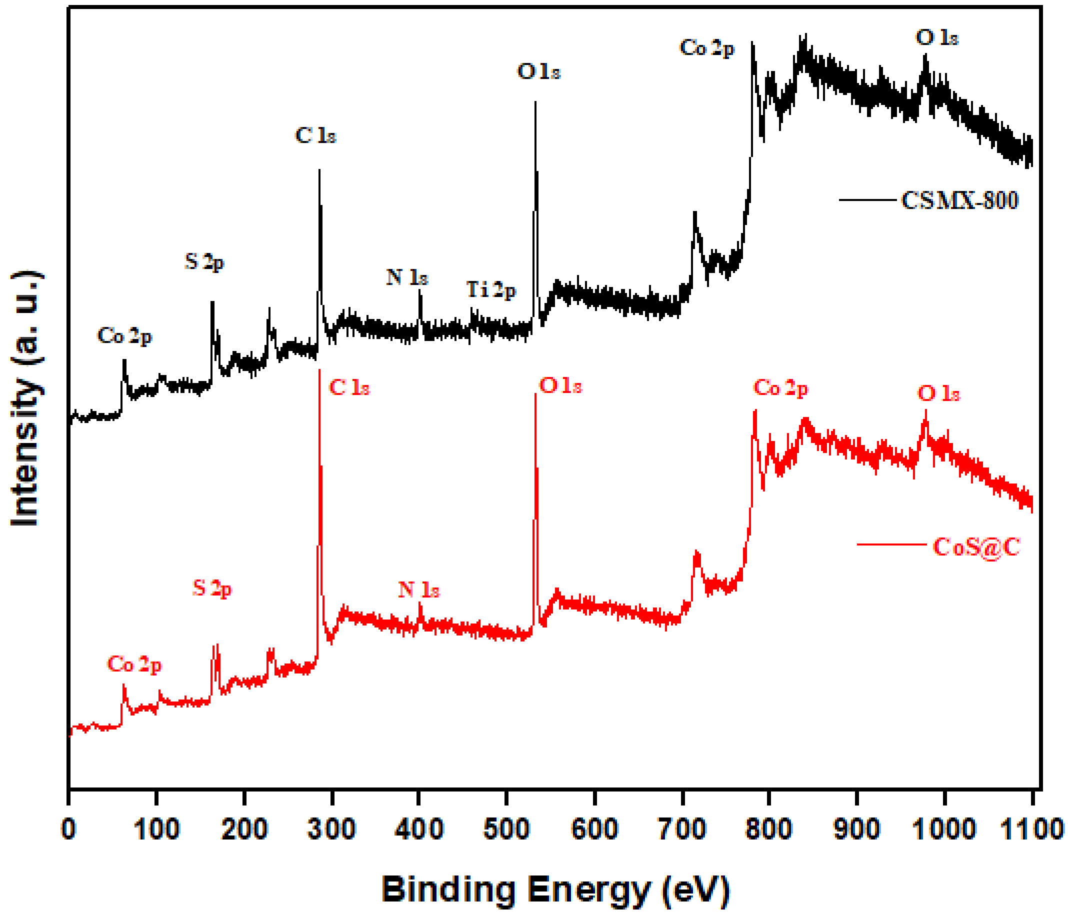

Fig. S4 XPS element survey spectrum of representative samples CSMX-800 and CoS@C.

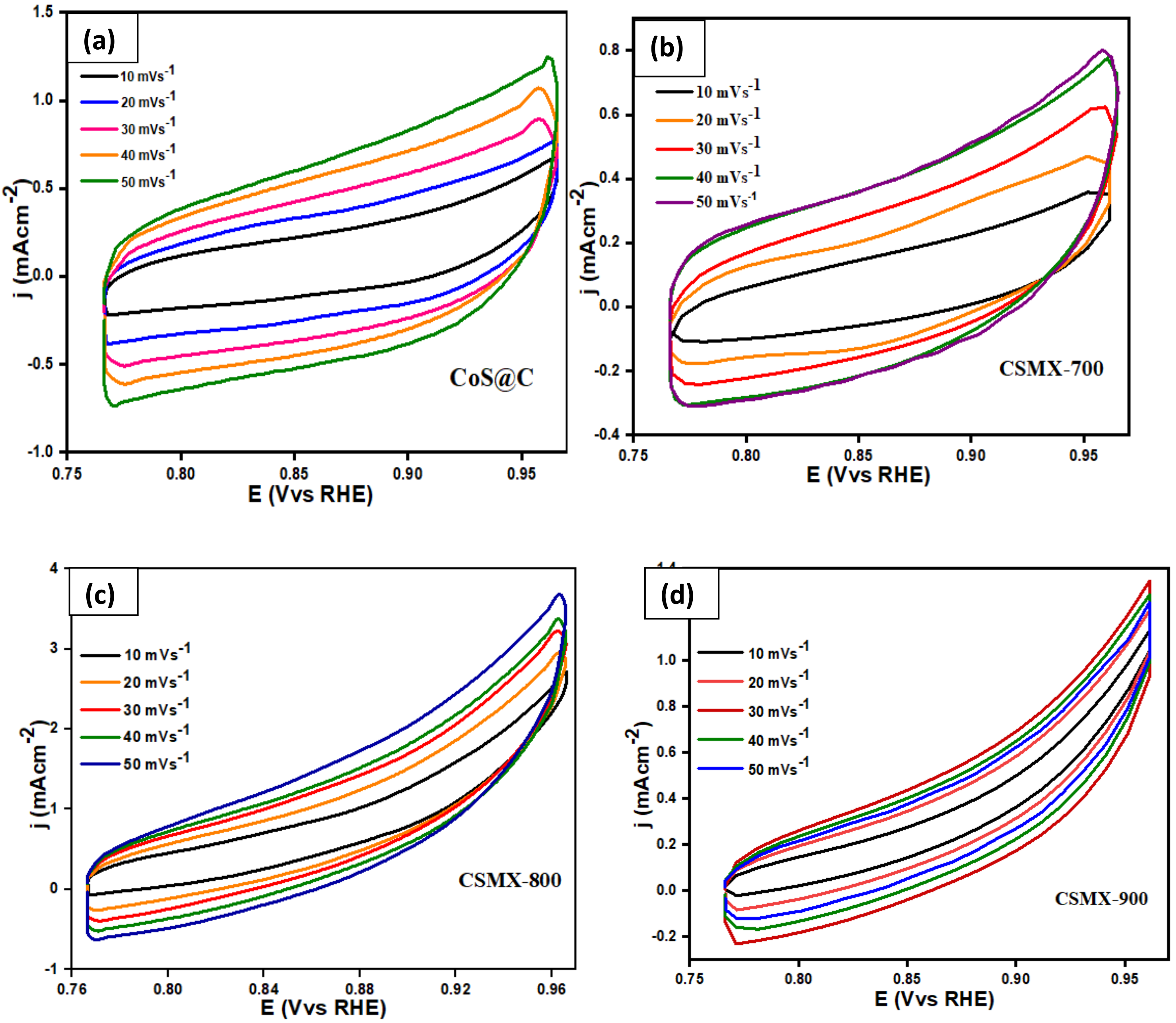

Fig. S5 Non-faradic region cyclic voltammogram at various scanning rates of (a) CoS@C, (b) CSMX-700, (c) CSMX-800 and (d) CSMX-900.

Table S1 Textural properties of studied samples

**Sample**	**CSMX-700**	**CSMX-800**	**CSMX-900**	**CoS@C**
**Surface area** (m^2 g^{-1})	160	166	172	211
**Total pore volume** (cm^3 g^{-1})	0.13	0.15	0.21	0.22

Table S2 Overpotential and Tafel slope values for OER and HER

**Electrocatalyst**	**OER overpotential value at10 mA cm^{-2} (mV vs. RHE)**	**Tafel slope (mV dec^{-1})**	**HER overpotential value at 10 mA cm^{-2} (mV vs. RHE)**	**Tafel slope (mV dec^{-1})**
**CSMX-700**	339	134	323	283
**CSMX-800**	257	95	190	103
**CSMX-900**	305	100	265	223
**CoS@C**	403	137	496	310

Table S3 C_{dl}, ECSA and R_f values of samples

**Electrocatalyst**	**C_{dl} (mF)**	**ECSA (cm^2)**	**R_f***
**CSMX-700**	0.011	0.55	7.78
**CSMX-800**	0.0237	1.185	16.76
**CSMX-900**	0.016	0.8	11.31
**CoS@C**	0.014	0.7	9.90

*Roughness factor R_f = ECSA/electrode's geometrical area.
